# Ethanol Extract of Yak-Kong Fermented by Lactic Acid Bacteria from a Korean Infant Markedly Reduces Matrix Metallopreteinase-1 Expression Induced by Solar Ultraviolet Irradiation in Human Keratinocytes and a 3D Skin Model

**DOI:** 10.3390/antiox10020291

**Published:** 2021-02-15

**Authors:** Heanim Park, Ji Won Seo, Tae Kyung Lee, Jae Hwan Kim, Jong-Eun Kim, Tae-Gyu Lim, Jung Han Yoon Park, Chul Sung Huh, Hee Yang, Ki Won Lee

**Affiliations:** 1 Department of Agricultural Biotechnology and Research Institute of Agriculture and Life Sciences, Seoul National University, Seoul 08826, Korea; km36583@naver.com (H.P.); Thinkbreaker@naver.com (J.W.S.); vluetk@snu.ac.kr (T.K.L.); kjhh720@naver.com (J.H.K.); jyoon@hallym.ac.kr (J.H.Y.P.); 2 Department of Food Science and Technology, Korea National University of Transportation, Jeungpyeong 27909, Korea; idonlike@gmail.com; 3 Department of Food Science & Biotechnology, Sejong University, Seoul 05006, Korea; tglim@sejong.ac.kr; 4 Advanced Institutes of Convergence Technology, Seoul National University, Suwon 16229, Korea; 5 Graduate School of International Agricultural Technology, Seoul National University, Pyeongchang 25354, Gangwon-do, Korea; cshuh@snu.ac.kr; 6 Center for Food and Bioconvergence, Seoul National University, Seoul 08826, Korea

**Keywords:** Yak-Kong, fermentation, lactic acid bacteria derived from infants, skin wrinkle, MMP-1, human keratinocytes, 3D skin model

## Abstract

Yak-Kong is a type of black soybean that is colloquially referred to as the “medicinal bean” and it elicits several beneficial effects that are relevant to human health, including attenuating the formation of skin wrinkles. It has previously been shown that soybean extracts elicit additional bioactivity that is fermented by lactic acid bacteria. In this study of lactic acid bacteria strains that were isolated from the stools of breast-feeding infants (<100 days old), we selected *Bifidobacterium animalis* subsp. Lactis LDTM 8102 (LDTM 8102) as the lead strain for the fermentation of Yak-Kong. We investigated the effects of LDTM 8102-fermented Yak-Kong on solar-ultraviolet irradiation (sUV)-induced wrinkle formation. In HaCaT cells, the ethanol extract of LDTM 8102-fermented Yak-Kong (EFY) effectively reduced sUV-induced matrix metalloproteinase-1 (MMP-1) secretion. The effect of EFY was superior to that of unfermented (UFY)- and Lactis KCTC 5854 (another *Bifidobacterium animalis* species)-fermented Yak-Kong. Additionally, EFY reduced sUV-induced MMP-1 mRNA expression and promoter activity, as well as the transactivation of AP-1 and phosphorylation of ERK1/2 and JNK1/2. Furthermore, EFY alleviated sUV-induced MMP-1 secretion, the destruction of the epidermis, and degradation of collagen in a three-dimensional (3D) skin culture model. EFY had a higher total polyphenol content and anti-oxidative activity than UFY. Twelve metabolites were significantly (≥2-fold) increased in Yak-Kong extract after fermentation by LDTM 8102. Among them, the metabolites of major isoflavones, such as 6,7,4′-trihydroxyisoflavone (THIF), exerted the reducing effect of MMP-1, which indicated that the isoflavone metabolites contributed to the effect of EFY on MMP-1 expression as active compounds. These findings suggest that EFY is a potent natural material that can potentially prevent sUV-induced wrinkle formation.

## 1. Introduction

Skin aging presents a significant challenge to humankind, and a wide range of research has been undertaken to address this problem. There are two main types of skin aging [1]. The first is intrinsic (chronological) aging, or natural aging over time, which is not affected by the environment. The second is extrinsic aging, or aging that is caused by the environment [2]. Solar ultraviolet irradiation is the primary contributor that is responsible for most cases of extrinsic aging [3]. Exposure to solar-ultraviolet irradiation (sUV) contributes to approximately 90% of overall changes to skin, in a process that is referred to as photoaging [4]. sUV irradiation generates reactive oxygen species (ROS), causing oxidative stress in skin cells, which leads to alterations in key signal transduction pathways, including the mitogen-activated protein kinase (MAPK) pathway [5]. 

Collagen, the primary structural component of the dermis, helps to maintain the integrity of skin tissue [6]. ROS activate the expression of the matrix metalloproteinase (MMP) family members through MAPK signaling [7], which results in the degradation of other components of the extracellular matrix, such as elastin, glycosaminoglycans, and interstitial collagen, within dermal connective tissue [8]. In the MMP family, MMP-1 is a major enzyme that contributes to wrinkle formation by breaking down collagen in the skin [9,10]. The expression and secretion of MMP-1 depends on transcription factors, such as activator protein-1 (AP-1), which is regulated by the phosphorylation of MAPK signaling, including extracellular-signal regulated kinase 1/2 (ERK1/2), c-Jun N-terminal kinase 1/2 (JNK1/2), and protein 38 (p38) [11,12,13].

Yak-Kong (*Seomok-tae*; Rhynchosia nulubilis), which is a type of black soybean, is colloquially referred to as the “medicinal bean”, and it is higher in flavonoids and amino acids when compared to other soybeans [14]. It has higher antioxidant activity than ordinary black soybeans and yellow soybeans [15,16]. Moreover, Yak-Kong elicits a number of reported beneficial effects, such as detoxification properties in human umbilical vascular endothelial cells [17], attenuating effects in the formation of skin wrinkles, and positive effects on elastase activity in dermal keratinocytes [18]. 

Microbial fermentation has long been used for various purposes in the food industry [19]. In recent years, fermentation strategies have been used to improve taste and preserve foods, as well as enhance the health benefits of the raw ingredients [19,20]. Soybeans that have undergone microbial fermentation exhibit increased concentrations of several peptides, free amino acids, and phenolic compounds, such as isoflavones, which results in anti-thrombotic and anti-cancer effects [21,22]. However, the beneficial effects of Yak-Kong following fermentation by microbes, such as lactic acid bacteria, have not been widely studied. Here, we investigated whether the beneficial effects of Yak-Kong on sUV-induced photoaging can be improved by fermentation with lactic acid bacteria. For the first time, we found that Yak-Kong extract that was fermented by lactic acid bacteria Bifidobacterium animalis subsp. Lactis LDTM 8102 (LDTM 8102) derived from an infant markedly inhibited sUV-induced MMP-1 expression in human keratinocytes and a three-dimensional (3D) skin model, leading to the reduction of sUV-induced epidermal destruction and collagen degradation. Additionally, the isoflavone metabolites 6,7,4′-trihydroxyisoflavone (THIF), 7,3′,4′-THIF, and 7,8,4′-THIF were specifically increased in Yak-kong that was fermented with LDTM 8102 as compared to non-fermented Yak-kong. Furthermore, these metabolites markedly reduced MMP-1 secretion in human keratinocytes. From these results, it was assumed that these compounds are active substances of LDTM 8102-fermented Yak-kong for reducing skin wrinkles.

## 2. Materials and Methods 

### 2.1. Reagents 

Dulbecco’s modified Eagle medium (DMEM) was purchased from Hycolne (Long, UT, USA). A solution of penicillin (10,000 IU) and streptomycin (10,000 µg/mL) was purchased from Mediatech (Manassas, VA, USA). Fetal bovine serum (FBS) was purchased from Seradigm (Radnor, PA, USA). 3-(4,5-dimethylthiazol-2-yl)-2,5-diphenyl tetrazolium bromide (MTT) was purchased from Affymetrix/USB (Cleveland, OH, USA). The MMP-1 antibody was purchased from R&D Systems (Minneapolis, MN, USA). Antibodies against phosphorylated extracellular-signal regulated kinase (ERK) 1/2 (Thr202/Tyr204), ERK1/2, total c-Jun N-terminal kinase (JNK) 1/2, and p38 were obtained from Santa Cruz Biotechnology (diluted to 1:1000; Santa Cruz, CA, USA). The antibody against p-p38 (pT180/pY182) was purchased from BD Bioscience (Franklin Lakes, NJ, USA), and phosphorylated stress-activated protein kinase/Jun-amino-terminal kinase (p-SAPK/JNK) (Thr183/Tyr185) antibody was purchased from Cell Signaling Biotechnology (Beverly, MA, USA). The protein assay reagent kits were purchased from Bio-Rad Laboratories (Hercules, CA, USA). 7,3′,4′- THIF, 6,7,4′-THIF, and 7,8,4′-THIF were purchased from Chromadex™ (Irvine, CA, USA).

### 2.2. Isolation and Identification of Probiotic Strains from Infant Feces

The Seoul National University Institutional Review Board approved the experimental protocol (no. 1605/003-006). We serially diluted 1 g infant feces 10-fold with phosphate-buffered saline to isolate probiotic strains from infant feces (PBS; pH 7.4; Mediatech, New York, USA). The mixture was spread onto de Man, Rogosa, and Sharpe (MRS) agar (Difco, Sparks, MD, USA), including 0.02% sodium azide (Sigma-Aldrich, St. Louis, MO, USA) and transoligosaccharide (TOS) propionate agar (Merck, Darmstadt, Germany). After incubating at 37 °C for 24–48 h in an anaerobic chamber (Coy Laboratories, Ann Arbor, MI, USA), single colonies that showed phenotypes for lactic acid bacteria and bifidobacteria were selected and subcultured under anaerobic conditions at 37 °C for 24 h in MRS broth that was supplemented with 0.05% L-cysteine-HCl (St. Louis, USA; MRSC, Seattle, USA). Gram staining and catalase tests were performed, as described previously [23]. Gram-stained bacteria were observed under an ECLIPSE Ci-L microscope (Nikon, Tokyo, Japan). The pure culture media in which single colonies were cultured were stored at −80 °C in 30% (*v/v*) glycerol solution and then sterilized with an autoclave at 121 °C for 15 min. prior to use. Bifidobacterium originating from the feces of 30 breast-feeding Korean an infant less than 100 days old were isolated. Among them, LDTM 8102 with excellent beta-glucosidase activity was selected as the main strain for fermentation of Yak-Kong. The LDTM 8102 were heat killed at 121 °C for 15 min. in order to produce dead cells (DC). We used Bifidobacterium animalis subsp. Lactis KCTC 5854 (KCTC 5854) as a reference control (REF) to compare strain-specific effects. KCTC 5854 used in this research were distributed from KCTC. 

### 2.3. Fermentation of Yak-Kong

**Figure 1:**
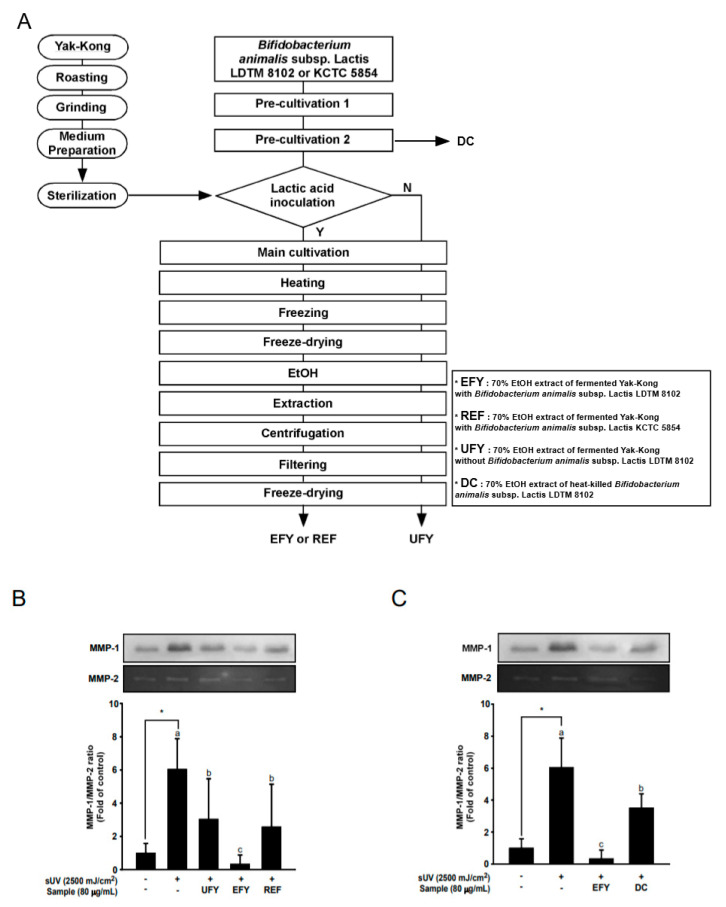
LDTM 8102 fermented Yak-Kong products extracted with 70% ethanol (EFY) inhibits solar-ultraviolet irradiation (sUV)-induced matrix metalloproteinase-1 (MMP-1) secretion to a greater extent than Unfermented Yak-Kong (UFY) and KTCT 5854 (another *bifidobacterium* strain) fermented Yak-Kong products extracted with 70% ethanol (REF). (**A**) Process for production of UFY, EFY, REF, and dead cells (DC). (**B**) Inhibitory effects of UFY, EFY, and REF on sUV-induced MMP-1 protein secretion. (**C**) Comparison of the inhibitory effects of EFY and DC on sUV-induced MMP-1 protein secretion in HaCaT cells. Data (*n* = 3) represent mean values ± SD. * significant difference (*p* < 0.05) between the control and sUV-induced control. Mean values with different letters indicate statistically significant differences among sUV-exposed groups (*p* < 0.05). Representative western blots from three independent experiments are shown (*n* = 3).

Figure 1A shows the process of fermenting Yak-Kong. Yak-Kong powder (100 mesh) was obtained from Naturetech (Jincheon, Korea), roasted, and then ground to a size smaller than a 60 mesh, and then dissolved into a medium consisting of distilled water (DW) that was adjusted to pH 6.5 with 1N NaOH. After sterilization, the lactic acid bacteria were inoculated into the medium. The strain used for fermentation, either LDTM 8102 or KCTC 5854, was pre-cultivated in MRSC at 37 °C for 24 h under anaerobic conditions. Subsequently, each pre-cultured strain was inoculated into the sterilized Yak-Kong dissolved medium to a final concentration of 1% (*v/v*), corresponding to approximately 1 × 10^6^ CFU/mL, and then fermented at 37 °C at 70 rpm for 48 h. The samples were collected for measurements of cell viability and pH during fermentation. After 48 h of fermentation, we placed the Yak-Kong in a hot water bath at 90 °C for 11 min. to kill the lactic acid bacteria and terminate the fermentation reaction. Prior to extraction, the fermented Yak-Kong products were stored at −80 °C overnight and then lyophilized. Freeze-dried LDTM 8102-fermented Yak-Kong products were extracted with 70% ethanol (EFY) for 2 h in a shaking incubator. Unfermented Yak-Kong products (UFY) and KCTC 5854-fermented Yak-Kong products (REF) were extracted under the same conditions. The supernatant was filter sterilized with a syringe filter (a membrane pore size of 0.22 μm: Younginfrontier, Seoul, Korea). Each extracted product was freeze-dried again and then dissolved in dimethyl sulfoxide (DMSO) for cell treatments.

### 2.4. Cell Culture and sUV Irradiation 

HaCaT cells, skin keratinocytes, were obtained from CLS Cell Lines Services (Heidelberg, Germany). They were cultured in DMEM with 10% (v/v) FBS and penicillin (100 IU)/streptomycin (100 µg/mL) in a 5% CO_2_ atmosphere at 37°C, as reported in many previous researches [24,25,26]. We seeded the cells with DMEM containing 10% FBS in a six-well plate at a density of 4.5 × 10^5^ cells per well to examine the anti-photoaging effects of EFY on HaCaT cells. The cells were cultured for 24 h, and the medium was changed to serum-free DMEM. After additional incubation at 37 °C for 24 h, the HaCaT cells were pretreated with serum-free medium with various concentrations (0–80 μg/mL) of the Yak-Kong samples for 1 h, followed by exposure to sUV at an intensity of 2500 mJ/cm^2^. The irradiation instrument for exposure to sUV was a UVA-340 lamp that was obtained from Q-Lab (Cleveland, OH, USA). The UVA-340 lamp most closely mimics natural sUV. The critical short wavelength UVC region under 295 nm is blocked, and peak emission occurs at 340 nm. The proportion of UVA and UVB radiation that was produced by the UVA-340 lamp was measured with a UV meter: UVA made up 94.5% of the total radiation, with UVB making up the other 5.5%. When considering that the average amount of UV that arrived on the ground in a day, so-called daylight UV, is 37.75 J/cm^2^ in New York City [27], we used solar UV with an intensity of 2000 or 2500 mJ/cm^2^, which is the similar extent of UV that is received when a human in New York City was exposed to daylight for about an hour.

### 2.5. Cell Viability

The MTT assay was performed to measure the effect of samples on the cell viability in HaCaT cells. The cells were seeded in 96-well plates at a density of 10 × 10^4^ cells per well and then incubated for 24 h in DMEM with 10% (*v/v*) FBS and penicillin (100 IU)/streptomycin (100 µg/mL) in a 5% CO2 atmosphere at 37°C. After then, the cells were additionally incubated in serum-free DMEM for 24 h, and different concentrations (20 to 80 μg/mL) of fermented Yak-kong were treated to the cells. One hour after pre-treatment of samples, the cells were exposed to 2500 mJ/cm^2^ sUV irradiation and then incubated for 48 h at 37 °C. Two hours after the addition of 10% (*v/v*) MTT solution to medium, the medium was removed and DMSO was added to allow formazan crystals to be fully dissolved. Absorbance was measured at 570 nm while using a microplate reader (Molecular Devices, CA, USA).

### 2.6. Western Blottin Assay

For MMP-1 protein detection, the media were harvested on ice 48 h after sUV irradiation (2500 mJ/cm^2^) and centrifuged for 10 min. at 18,620× *g*. After the cell culture media were removed, the cells were lysed with a RIPA lysis buffer consisting of 10 mM Tris (pH 7.5), 150 mM NaCl, 5 mM ethylenediamunetetraacetic acid, 0.1% Triton X-100, 1 mM dithiothreitol, 0.1 mM phenylmethylsulfonyl fluoride, 10% glycerol, and a protease inhibitor cocktail tablet (Gendepot, TX, USA). The protein concentration of the media or lysates was measured with protein assay reagent kits following the manufacturer’s instructions. The proteins in the media or lysates were electrophoretically separated by 10% sodium dodecyl sulfate (SDS)–polyacrylamide gel electrophoresis (PAGE), and then transferred to Immobilon-P polyvinylidene fluoride or polyvinylidene difluoride membranes (Merck Millipore, Darmstadt, Germany). The membrane was blocked in 5% skim milk for 1 h and then incubated at 4 °C overnight to attach the specific primary antibody. Primary antibodies were diluted at a 1:1000 ratio. After attaching the HRP-conjugated secondary antibody (Life Technologies, Waltham, MA, USA), we visualized the protein bands on the membrane using a chemiluminescence detection kit (GE Healthcare, Little Chalfont, UK).

### 2.7. Gelatin Zymography

MMP-2 was used as a loading control for MMP-1 western blots, because the protein levels of the gelatinase MMP-2 are not affected by sUV irradiation [28,29]. Gelatin zymography was performed with 12% polyacrylamide gel containing 0.1% (*w/v*) gelatin as a substrate for MMP-2. The protein samples were mixed with loading buffers consisting of 10% SDS, 25% glycerol, 0.25 M Tris (pH 6.8), and 0.1% bromophenol blue. Mixed samples were run on 12% SDS-PAGE without denaturation. The loaded gels were washed with renaturing buffer (Life Technologies) for 1 h and then incubated in developing buffer (Life Technologies) at 37 °C for 16 h. After the enzyme reaction was finished, the gels were stained with 0.5% Coomassie Brilliant Blue with 10% acetic acid.

### 2.8. RNA Preparation and Real-Time Quantitative PCR

HaCaT cells were treated with the indicated concentrations of samples and stimulated with 2500 mJ/cm^2^ sUV. Twenty-four h after the sUV irradiation, RNA was isolated with RNAiso Plus (Takara Bio, Shiga, Japan), according to manufacturer’s instructions. The purity and concentration of the RNA samples were measured with a NanoDrop ND-2000 spectrophotometer (Thermo Fisher Scientific, Waltham, MA, USA). A sample of 1 µg RNA was used for reverse transcription with oligo-dT primers using a PrimeScript™ 1st strand cDNA synthesis kit (Takara Bio, Shiga, Japan). Real-time quantitative PCR was performed with iQ SYBR (Bio Rad Laboratories, CA, USA) with a CFX Connect™ Real-Time PCR Detection System (Bio-Rad Laboratories, CA, USA). The primers were denatured at 95 °C for 3 min., and we conducted PCR amplification by repeating 44 cycles of 95 °C for 10 s, 60 °C for 30 s, and 72 °C for 30 s. PCR was performed and cDNA was probed with the following primers: MMP-1 forward (5′-CCC CAA AAG CGT GTG ACA GTA-3′), MMP-1 reverse (5′-GGT AGA AGG GAT TTG TGC G-3′); glyceraldehyde 3-phosphate dehydrogenase (GAPDH) forward (5′-GAG TCA ACG GAT TTG GTC GT-3′), a d GAPDH reverse (5′-TTG ATT TTG GAG GGA TCT CG-3′). GAPDH was used as an internal control.

### 2.9. Luciferase Reporter Gene Assay

The lentiviral expression vector pGF-AP1-mCMV-EF1-Puro was obtained from System Biosciences (Palo Alto, CA, USA) and packaging vectors containing pMD2.0G and psPAX were purchased from Addgene (Cambridge, MA, USA). pGF-MMP-1-mCMV-EF1-puro vector was constructed with a cloning MMP-1 promoter to pGreenFire (pGF1) plasmid by Sung Keun Jung (Kyungpook National University, Dae-Gu, Korea) [30]. We transfected HEK293T cells to pGF-MMP-1-mCMV-EF1-puro and pGF-AP1-mCMV-EF1-Puro vectors, together with packaging vectors pMD2.0G and psPAX, respectively, using a jetPEI according to the manufacturer’s instructions. After transfection, the transfection medium was changed, and the cells were incubated for 36 h. The viral particles were filtered through a 0.45 µm syringe filter, and then the filtrates were combined with 8 µg/mL polybrenes (Merck Millipore, MA, USA) and then infected into 60% confluent HaCaT cells overnight. After more than 36 h of puromycin (2 mg/mL) treatment, only infected cells were selected, and the cell culture medium was replaced with fresh medium. The transfected cells were used in the luciferase reporter gene assay. AP-1 luc HaCaT cells and MMP-1 luc HaCaT cells were cultured for 48 h and then starved to serum-free DMEM for 24 h. Afterwards, they were pretreated for 1 h with the indicated concentrations of samples, followed by irradiation with 2500 mJ/cm^2^ sUV. AP-1 and MMP-1 luc HaCaT cells were lysed 6 h and 8 h after sUV irradiation, respectively. MMP-1 promotor activity and AP-1 transactivation were measured with a luciferase assay kit (Promega, WI, USA), as described by the manufacturer.

### 2.10. Assay of DPPH Scavenging Activity

In each well of a 96-well plate, 6.7 µL of each test sample or standard antioxidant solution (100 µg/mL ascorbic acid) and 193.3 µL DPPH solution were added. The plate was shaken vigorously and then left to stand in the dark at room temperature for 30 min. After incubation, the absorbance of the resulting solution was measured at 517 nm with a microplate reader (Molecular Devices, CA, USA). The total antioxidant capacity was indicated as the vitamin C equivalent antioxidant capacity (VCEAC).

### 2.11. 3D Artificial Skin Culture and Treatments

A Neoderm-ED 12-well plate was purchased from Tego Science (Seoul, Korea). Subsequently, three-dimensional (3D) artificial skin tissue was incubated at 37 °C and 5% CO_2_ with a dedicated medium being provided by Tego Science. For sUV-induced photoaging, 3D artificial skin tissue was pretreated with different concentrations (20 to 80 μg/mL) of EFY for 1 h and then exposed to 2000 mJ/cm^2^ sUV twice a day for two days at 2 h intervals. Twenty-four h after the last sUV irradiation, the medium was harvested for the detection of MMP-1 expression and the artificial skin tissue was fixed in 4% formaldehyde solution for Masson’s trichrome staining.

### 2.12. Immunocytochemistry

We performed Masson’s trichrome staining to evaluate the collagen content of the dermis and the epidermis thickness in 3D artificial skin tissue. A total of 24 h after the last sUV irradiation, artificial skin tissue was fixed with 4% formaldehyde solution, and tissue staining was performed by Tego Science. Briefly, fixed 3D artificial skin tissue was embedded and de-paraffinized, then stained with hematoxylin for 5 min. The slides were washed and stained with biebrich scarlet and acid fuchsin. Next, the slides were stained for 10 min. in phosphomolybdic-phosphotungstic acid and for 5 min. in aniline blue to stain the collagen tissue. They were washed and incubated for 15 min. in 1% acetic acid, dehydrated, and then washed. The slides of the 3D artificial skin tissue were examined and photographed at 400× magnification with an Olympus AX70 light microscope (Tokyo, Japan).

### 2.13. Assay of Total Phenolic Phytochemical Content

Ten µL sample or standard (100 µg/mL gallic acid), 10 µL Folin & Ciocalteu’s phenol reagent (Sigma-Aldrich, St. Louis, MO, USA), and 80 µL distilled water were added in each well of a 96-well plate and then shaken for 6 min. After the reaction, 100 µL 7% (*w/v*) sodium carbonate solution was placed into each well and shaken for 90 min. at room temperature. As the reaction proceeded and the solution turned blue, absorbance was measured at a wavelength of 750 nm with a microplate reader. The total phenolic content was estimated from the calibration curve as the standard gallic acid equivalent (GAE).

### 2.14. Ultra Performance Liquid Chromatography and Time-of-Flight Mass Spectrometry (UPLC−TOF-MS) Analysis

The EFY and UFY samples were lyophilized and then ground to a powder for further analysis. The powdered samples were extracted with 80% methanol for 2 h and centrifuged at 850× *g* for 10 min. The supernatant was filtered through a 0.22 μm nylon syringe filter. The supernatant was subjected to UPLC Q-TOF MS analysis by the National Instrumentation Center for Environmental Management (NICEM), Seoul, Korea. The samples (2 μL) were directly injected into a 1290 Infinity ultra-high-pressure liquid chromatograph coupled with a dual Agilent jet stream electrospray ionization (ESI; Agilent, CA, USA) source to a Triple TOF 5600 system (AB SCIEX, Concord, ON, Canada). The column temperature was maintained at 45 °C. All of the samples had been kept at 4 °C during the data collection period. 

### 2.15. Metabolomics Data Processing

The metabolites were identified by searching the metabolomics databases Human Metabolome Database (HMDB). The metabolite profiling data of fermentation and non-fermentation samples were imported into Metaboanalyst 4.0 (http://www.metaboanalyst.ca (accessed on 15 Decmber 2020)) for multivariate statistical analysis and visualization. Partial least square discriminant analysis (PLS-DA) was performed and variable importance in projection (VIP) score was obtained in order to find the potential discriminatory metabolites for clustering samples based on metabolic profiles. The top 25 metabolites were regarded as the potential components that played a key role in the differentiation between EFY and UFY samples. The relative abundance of the selected metabolites was presented by heatmap analysis to visualize the differences between EFY and UFY samples. The volcano plot in Metaboanalyst was performed to represent the significant increased metabolites in EFY with a threshold of *p*-value of 0.05 (*Y*-axis) and a fold change of 2 (*X*-axis) as compared to UFY. We used high-performance liquid chromatography (HPLC) by NICEM for the quantification of metabolites.

### 2.16. Statistical Analyses

The results are presented as means ± standard deviations (SDs). Differences between the control group and the sUV-exposed group were assessed with Student’s t test. Differences between the sUV-exposed groups were verified by one-way analysis of variance (ANOVA) with Duncan’s multiple range test. The data were statistically analyzed with SPSS Statistics ver. 23.0 (IBM, Armonk, NY, USA). *p* < 0.05 was considered to be statistically significant.

## 3. Results 

### 3.1. EFY More Effectively Suppresses sUV-Induced MMP-1 Secretion Than UFY and REF

EFY, UFY, and REF samples were generated following the process broadly described in Figure 1A to examine whether EFY, the ethanol extract of Yak-Kong fermented by LDTM 8102, effectively reduces MMP-1 expression. sUV irradiation of HaCaT Cells significantly increased MMP-1 protein secretion into the culture media, as compared to the sUV-unexposed control. EFY inhibited sUV-induced MMP-1 protein secretion more effectively than UFY (Figure 1B), and reduced MMP-1 secretion to a greater extent than REF (Figure 1B). Additional tests were conducted in order to elucidate whether the reduction in MMP-1 protein secretion that was induced by EFY was due to DC or metabolites produced by fermentation. The results showed that the EtOH extract of dead cells inhibited the sUV-induced MMP-1 protein secretion. However, the effect of DC extract was much smaller than that of EFY (Figure 1C). Taken together, these results suggest that EFY is a potent inhibitor of sUV-induced MMP-1 protein secretion. The metabolites that are produced by fermentation appear to contribute to the effects of EFY on MMP-1 secretion

### 3.2. EFY Reduces sUV-Induced MMP-1 Protein Secretion and mRNA Expression

**Figure 2:**
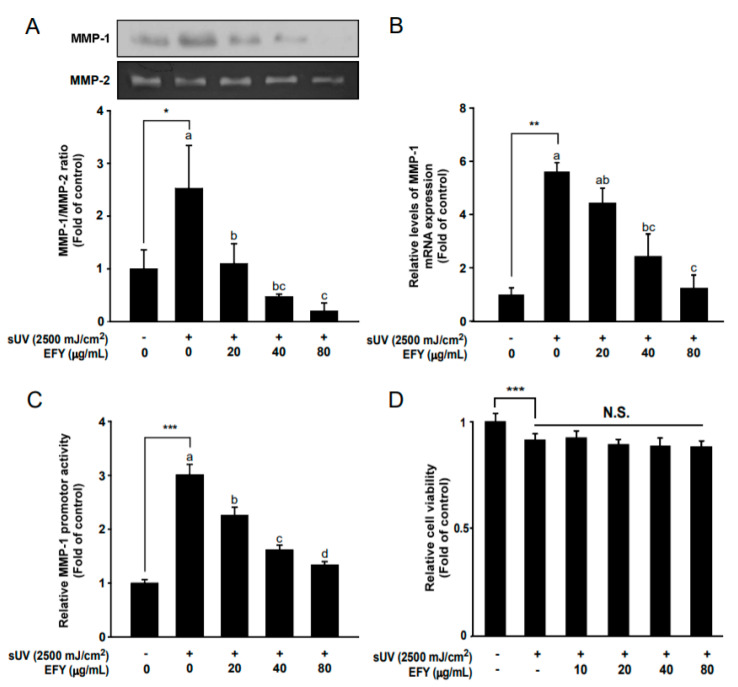
EFY inhibits sUV-induced MMP-1 protein secretion and reduces mRNA transcription via the suppression of MMP-1 promoter activity. (**A**) Inhibitory effects of EFY on sUV-induced MMP-1 protein secretion and Quantification of the effects of EFY on sUV-induced MMP-1 protein secretion. (**B**) Reduction in sUV-induced MMP-1 mRNA expression due to EFY. (**C**). MMP-1 promoter activity in HaCaT cells. (**D**). Cell viability was measured using a MTT assay. Data (*n* = 3) are mean values ± SD. *** significant difference (*p* < 0.001), ** (*p* < 0.01) and * (*p* < 0.05) between the control and sUV-exposed control. N.S., not significant. Mean values with different letters indicate statistically significant differences among sUV-exposed groups (*p* < 0.05). Representative western blots from three independent experiments are shown (*n* = 3).

In order to determine how EFY regulates MMP-1 protein expression, its effects on sUV-induced MMP-1 expression were examined in terms of protein secretion and at the transcriptional level. EFY significantly reduced sUV-induced MMP-1 protein secretion at 40 and 80 µg/mL (Figure 2A). sUV irradiation also increased the mRNA expression of MMP-1 by around six-fold when compared to the control (Figure 2B). EFY significantly reduced sUV-induced MMP-1 mRNA expression at the same concentrations (40 and 80 µg/mL). In addition, sUV irradiation increased the MMP-1 promoter activity around three-fold compared to the control, while EFY reduced sUV-induced MMP-1 promoter activity in a dose-dependent manner (20, 40, and 80 µg/mL; Figure 2C). The results of the MTT assay revealed that, although sUV irradiation slightly affected on cell viability up to about 9%, there were no significant changes in cell viability by EFY sample treatments (Figure 2D).

### 3.3. EFY Suppresses sUV-Induced AP-1 Transactivation in HaCaT Cells

**Figure 3:**
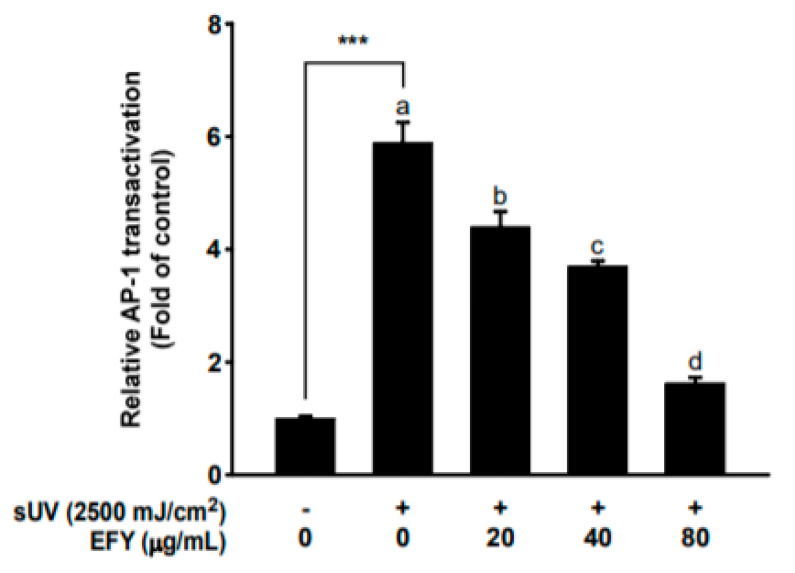
EFY suppresses sUV-induced transactivation of AP-1 in HaCaT cells. Data (*n* = 3) are mean values ± SD. *** significant difference (*p* < 0.001) between the control and sUV-exposed control. Mean values with different letters indicate statistically significant differences among sUV-exposed groups (*p* < 0.05).

The AP-1 transcription factor is a key regulator of sUV-induced transcription of MMP-1 [31]. A luciferase reporter gene assay was performed using AP-1 luc vector-transfected HaCaT cells in the presence or absence of EFY (20, 40, and 80 µg/mL) to investigate whether EFY suppresses sUV-induced AP-1 transactivation in HaCaT cells. sUV irradiation effectively induced AP-1 transactivation up to approximately six-fold as compared to the control (Figure 3). EFY reduced sUV-induced AP-1 transactivation in HaCaT cells in a dose-dependent manner, and (80 µg/mL) reduced the AP-1 transactivation levels by 72% when compared to the sUV-induced control (Figure 3).

### 3.4. EFY Downregulates sUV-Induced Phosphorylation of ERK1/2 and JNK1/2 in HaCaT Cells 

**Figure 4:**
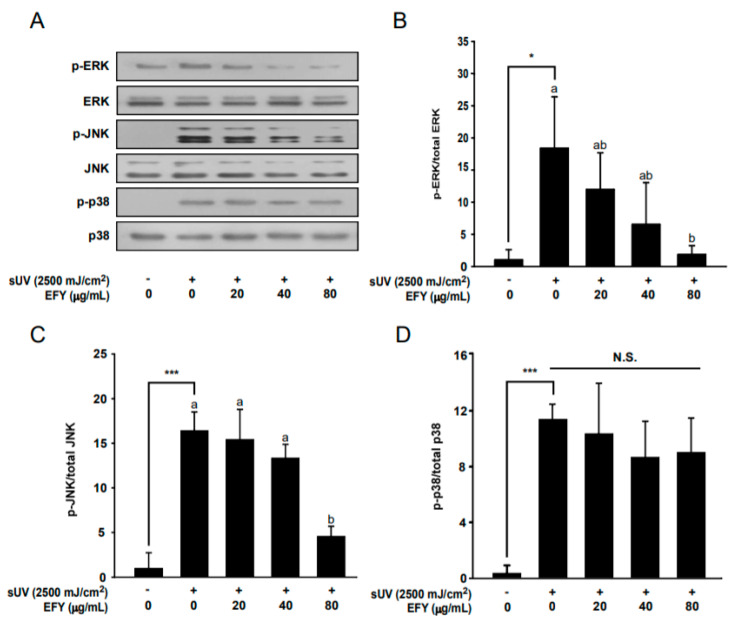
EFY inhibits sUV-induced phosphorylation of ERK1/2 and JNK1/2 but not p38. (**A**) EFY reduces sUV-induced phosphorylation of ERK1/2 and JNK1/2 in HaCaT cells. (**B**–**D**) Quantification of sUV-induced phosphorylated MAPKs in HaCaT cells without the presence and absence of EFY. Each phosphorylated form of the MAPK proteins was normalized to the corresponding total form of the proteins. Data (*n* = 3) are mean values ± SD. * significant difference (*p* < 0.05) and *** significant difference (*p* < 0.001) between the control and sUV-exposed control. N.S., not significant. Mean values with different letters indicate statistically significant differences among sUV-exposed groups (*p* < 0.05). Representative western blots from three independent experiments are shown (*n* = 3).

ERK1/2, JNK1/2, and p38 phosphorylation was examined by Western blot to investigate whether EFY regulates sUV-induced activation of MAPKs (upstream signaling proteins of AP-1). Treatment with 80 µg/mL EFY significantly reduced sUV-induced phosphorylation of ERK1/2 (Figure 4A,B) and JNK1/2 (Figure 4A,C) in HaCaT cells. However, EFY did not alter sUV-induced phosphorylation of p38 at 20, 40, or 80 µg/mL (Figure 4A,D).

### 3.5. EFY Prevents sUV-Induced Destruction of the Epidermis and Degradation of Collagen in a 3D Culture Skin Model

**Figure 5:**
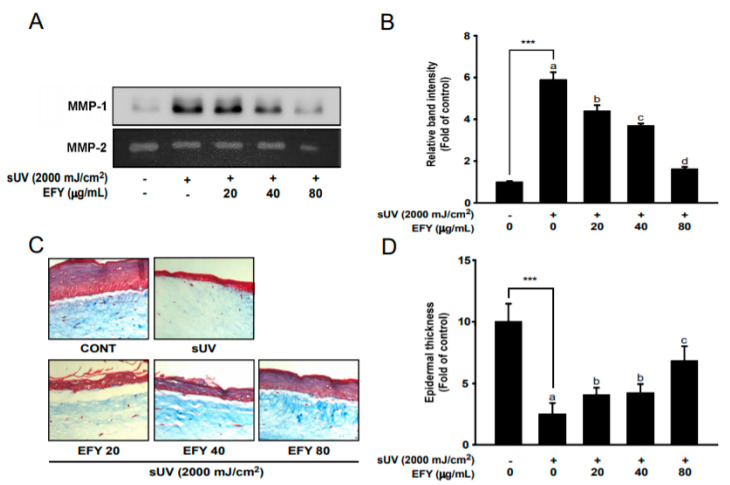
EFY attenuates sUV-induced MMP-1 secretion and skin damage in a three-dimensional (3D) skin culture model. (**A**,**B**) Inhibitory effects of EFY on MMP-1 protein secretion in 3D artificial skin. MMP-2 expression was used for the normalization of MMP-1. Data (*n* = 3) are mean values ± SD. (**C**). Masson’s trichrome staining to visualize collagen fiber and epidermal thickness. (**D**) Quantification of epidermal thickness. Data (*n* = 5) are mean values ± SDs. *** significant difference (*p* < 0.001) between the control and sUV-exposed control. Mean values with different letters indicate statistically significant differences among sUV-exposed groups (*p* < 0.05). Representative western blots from three independent experiments are shown (*n* = 3).

We exposed 3D artificial skin tissue to sUV (2000 mJ/cm^2^) to induce changes that are associated with photoaging, including the destruction of the epidermis and degradation of collagen, in order to further investigate the anti-photoaging effects of EFY. As in HaCaT cells, EFY significantly reduced sUV-induced MMP-1 protein secretion in 3D artificial skin tissue in a dose-dependent manner (Figure 5A,B). The results of Masson’s trichrome staining showed that the epidermis was destroyed and collagens (stained blue) were degraded by the irradiation (Figure 5C). The thickness of the epidermis of the sUV-treated 3D culture artificial skin was considerably reduced, and EFY effectively recovered the sUV-induced reduction in epidermal thickness up to 64% at 80 µg/mL (Figure 5D).

### 3.6. EFY Has Higher Antioxidant Activity and Total Phenolic Content Than UFY 

**Figure 6:**
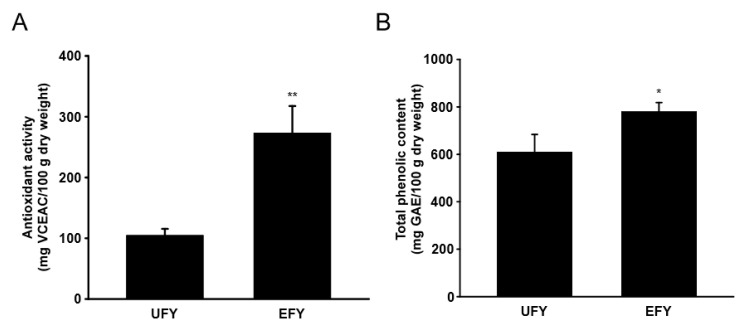
EFY has higher antioxidant activity and total phenolic content than UFY. (**A**) Antioxidant activity of EFY vs. UFY. The values were estimated as mg VCEAC per 100 g dry weight. (**B**) Total phenolic content of EFY vs. UFY. The values are mg gallic acid equivalent (GAE) per 100 g dry weight. Data (*n* = 3) are mean values ± SD. * significant difference (*p* < 0.05) and ** (*p* < 0.01) between UFY and EFY groups.

The antioxidant activity of UFY and EFY at a concentration of 1 mg/mL was examined with a DPPH radical scavenging assay because sUV irradiation can induce MMP-1 expression via the generation of ROS [5,7]. EFY had more antioxidant activity (273 mg VCEAC/100 g dry weight) than UFY (104 mg VCEAC/100 g dry weight; Figure 6A). We also examined the total phenolic content of EFY and UFY because a range of phenolic compounds exist in Yak-Kong [16]. The total phenolic content of EFY (779 mg GAE/100 g dry weight) was higher than that of UFY (609 mg GAE/100 g dry weight; Figure 6B). 

### 3.7. Comparison of Yak-Kong Metabolites before and after Fermentation

**Figure 7:**
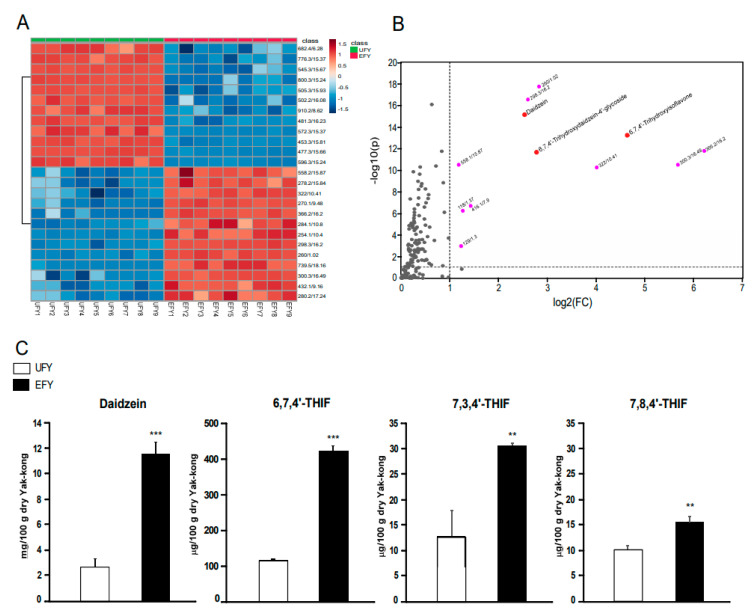

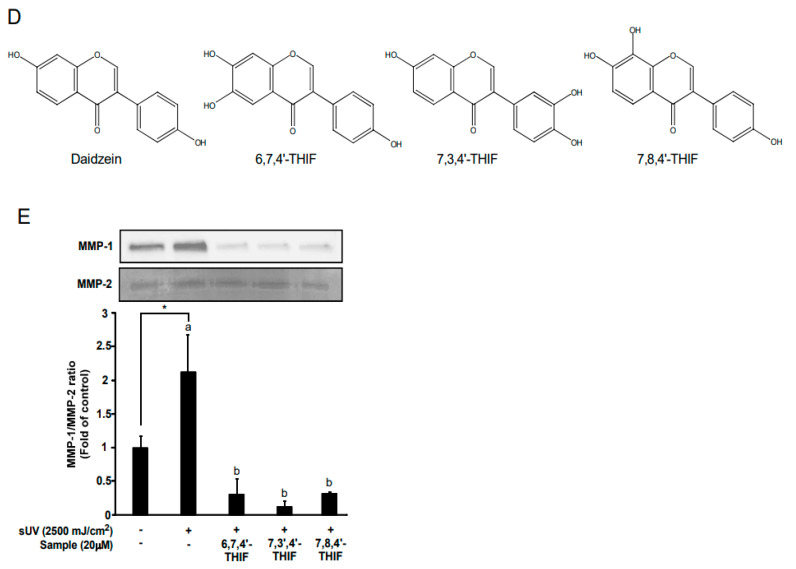
The main isoflavone metabolites of EFY exert the inhibitory effects of MMP-1 secretion. (**A**) Heatmap analysis of metabolite profiling of fermented and unfermented Yak-kong. The top 25 ranking metabolites (increased or decreased) were selected according to VIP values and presented against EFY and UFY as heat map. (**B**) The volcano plot for major metabolites increased in EFY. Twelve metabolites are shown with a difference of more than twice (*p* < 0.05) between EFY and UFY. (**C**) Quantification of daidzein, 6,7,4′-THIF, 7,3′,4′-THIF, and 7,8,4′-THIF before and after fermentation. Data (*n* = 3) are mean values ± SD. ** significant difference (*p* < 0.01) and *** (*p* < 0.001) between UFY and EFY. (**D**) Molecular structures of daidzein, 6,7,4′-THIF, 7,3′,4′-THIF, and 7,8,4′-THIF. (**E**). Inhibitory effects of 6,7,4′-THIF, 7,3′,4′-THIF, and 7,8,4′-THIF on sUV-induced MMP-1 protein secretion in HaCaT cells. The data represent mean values ± SD (*n* = 3). * significant difference (*p* < 0.05) between the control and sUV-induced control. Mean values with different letters indicate statistically significant differences among sUV-exposed groups (*p* < 0.05). Representative western blots from three independent experiments are shown (*n* = 3).

We conducted metabolite profiling of EFY using UPLC-QTOF-MS to find the fermented Yak-kong metabolites which may contribute to the beneficial effect of EFY on MMP-1 secretion. Of a total of 234 metabolites, 25 top-ranking metabolites (increased or decreased) taking into account the VIP values that were obtained from PLS-DA were selected and displayed in the heatmap plot (Figure 7A). The volcano plot showed the 12 most increased metabolites in EFY, with the fold change ≥ 2 and a statistical significance (*p* < 0.05) as the threshold (Figure 7B). Among these, daidzein and the daidzein metabolites 6,7,4′-THIF-4′-glycoside and 6,7,4′-THIF ranked high. Quantitative analysis using HPLC-MS showed that the concentrations of daidzein and 6,7,4′-THIF increased approximately four times each in EFY when compared to UFY. The concentrations of 7,3′,4′-THIF and 7,8,4′-THIF increased 2.4-fold and 1.4-fold in EFY, respectively, as compared to in UFY (Figure 7C). In Figure 7D, the molecular structures of daidzein, and its metabolites 6,7,4′-THIF, 7,3′,4′-THIF, and 7,8,4′-THIF were presented (Figure 7D). Remarkably, all of these three daidzein metabolites, 6,7,4′-THIF, 7,3′,4′-THIF, and 7,8,4′-THIF markedly suppressed the secretion of sUV-induced MMP-1 in HaCaT cells (Figure 7E).

## 4. Discussion

ROS are the primary mediators of sUV-induced photoaging [32]. When ROS are excessively generated by exposure to sUV, oxidative stress leads to transient and permanent DNA damage and the subsequent activation of the AP-1 protein, which results in the induction of MMP expression. This causes collagen breakdown, as well as wrinkle formation and inflammation in the skin tissue [33,34]. Previous studies have reported that antioxidants, such as retinoid, vitamin C, and polyphenols, improve skin wrinkles by blocking the action of ROS [35]. The antioxidant activity of Yak-Kong is superior to that of both ordinary black soybeans and yellow soybeans [16]. In addition, EFY exhibits significantly greater antioxidant activity than UFY (Figure 6A). We postulate that the anti-photoaging effects of EFY occur through the inhibition of sUV-induced MMP-1 protein secretion, which may be attributable to the improved ROS scavenging effects of EFY. However, the results from the DPPH radical scavenging assay are insufficient for explaining the high antioxidant activity of EFY. Further investigation as to whether EFY can reduce sUV-induced cellular ROS using other approaches, such as the 2′,7′ –dichlorofluorescin diacetate (DCF-DA) method, are needed to support our hypothesis.

During fermentation, macromolecules are broken down into small molecules by microbes, such as lactic acid bacteria; these small molecules are more easily absorbed and more effectively elicit bioactivity, including anti-photoaging effects [36,37,38]. We observed that the EtOH extract of dead cells inhibited the sUV-induced MMP-1 protein secretion; however, the effect of DC extract was much smaller than that of EFY (Figure 1C). These results indicate that EFY contains metabolites that are produced by the enzyme activity of lactic acid bacteria during fermentation and these metabolites may contribute to the beneficial effect of EFY on sUV-induced MMP-1 secretion. We conducted metabolite profiling of EFY and UFY using UPLC-QTOF-MS in order to investigate the changes in the composition of EFY during fermentation. Of the metabolites that undergo modification during fermentation of Yak-Kong by LDTM 8102, a total of 234 were identified using a human metabolite database [39,40]. Among them, twelve metabolites were observed as the most increased metabolites after fermentation with more than two-fold changes with statistical significance. The twelve increased metabolites can be classified into four different groups: phenolics (7,7′-dihydroxy-6,8′-bicoumarin and (Z)-4′,6-dihydroxyaurone 6-glucoside), flavonoids (daidzein, 6,7,4′-THIF, and 6,7,4′-THIF-4′-glucoside), lipids (xi-10-hydroxyoctadecanoic acid and ricinoleic acid), and others (cortolone, physalin I, pyroglutamic acid, glucose 6-phosphate, and succinic acid).

Phenolic compounds are known for the ameliorating effects of skin aging and skin damage, including wounds and burns [41]. Flavonoids are also well known for the beneficial effects on skin health, such as anti-wrinkle and anti-inflammatory effects. In particular, isoflavones are abundant flavonoids of soybeans exerting the beneficial effects on skin health [42,43]. Many previous studies have reported on the beneficial effects of soy isoflaovnes, including daidzein on skin health [44,45,46]. Soy isoflavones, such as daidzein, were metabolized during fermentation and isoflavone metabolites, such as 6,7,4′-THIF, 7,3′,4′-THIF, and 7,8,4-THIF, are produced [47]. Interestingly, in the current study, we observed 6,7,4′-THIF as the top three increased metabolite in EFY. Additionally, 7,3′,4′-THIF and 7,8,4-THIF, other daidzein metabolites, were also increased in EFY when compared to UFY. Our group previously reported that 6,7,4′-THIF, 7,3′,4′-THIF, and 7,8,4′-THIF had beneficial effects on skin health, such as wrinkle formation and cancer promotion, through their distinctive target proteins in various skin cell lines [48,49,50]. 6,7,4′-THIF suppressed sUV-induced MMP-1 in human dermal fibroblasts by directly targeting the Protein Kinase C α protein [49]. 7,3′,4′-THIF suppressed UVB-mediated skin cancer promotion in JB6+ P mouse epidermal cell lines by directly targeting MAPK kinase 4 and MAPK kinase 8 proteins [48]. 7,8,4′-THIF reduced UVB-induced MMP-1 expression in a human skin equivalent model by directly targeting Protein Kinase C ι protein [50]. These target proteins of each isoflavone metabolites have been determined to play a major role in skin wrinkle signal pathways [51,52,53]. In this study, we found that these three daidzein metabolites effectively suppressed sUV-induced MMP-1 in HaCaT Cells (Figure 7E).

Collectively, we can conclude that the beneficial effect of EFY on skin wrinkle may be, at least in part, attributed to the integrating effects of these isoflavone metabolites, including daidzein, 6,7,4′-THIF, 7,3′,4′-THIF, and 7,8,4′-THIF, increasing during fermentation process. Looking at the quantitative amount present in fermented Yak-kong by LDTM8102, daidzein may be a primary contributor among isoflavones to the effect of EFY. Interestingly, the concentration of 6,7,4′-THIF was approximately 10-fold the concentrations of the other daidzein metabolites, 7,3′,4′-THIF and 7,8,4-THIF. Therefore, although these three compounds were increased after fermentation and exerted similar effects on sUV-induced MMP-1 secretion, it can be reasonably assumed that 6,7,4′-THIF contributed more to the beneficial effect of EFY on skin wrinkle as compared to 7,3′,4′-THIF and 7,8,4-THIF.

In the present study, we found that EFY was superior at reducing sUV-induced MMP-1 secretion to REF (Figure 1B). LDTM 8102 was isolated from the stool of a Korean infant (less than 100 days old), whereas KCTC 5854 originated from yogurt (fermented milk). Therefore, although they both belong to *Bifidobacterium animalis* subsp. Lactis, the difference between these two lactic acid bacteria may be attributed to either their different microbial characteristics or the different kinds of metabolites that are produced by different microbial enzymes. 

We previously conducted the API ZYM (API) assay at the stage of selecting the suitable lactic acid bacteria for fermentation of Yak-Kong. The API is a simple and rapid screening system for identifying bacterial enzyme profiles. The results showed that our strain (LDTM8102) was one of the high ranked strains with high activity of glycoside hydrolase enzymes (i.g. α and β-glucosidase, α and β-galactosidase) as compared to other strains in *Bifidobacterium* species. Daidzein is known to be hydrolyzed to 6,7,4′-THIF, 7,3,4′-THIF, and 7,8,4′-THIF by glycosidic enzymes during soybean fermentation [54,55]. Thus, although we did not conduct metabolic enzyme profiling for direct comparison with KCTC 5854, since KTCT5854 is also one of the strains of *Bifidobacterium* species, it can be assumed that the higher glycosidic enzyme activity of LDTM8102 primarily contributed to the production of higher amounts of 6,7,4′-THIF, 7,3,4′-THIF, and 7,8,4′-THIF from daidzein in Yak-Kong.

In addition to the glycosidic enzymes, other enzymes, lipase (e.g., esterase, lipase) and amidase (e.g., leucinearylamidase, valinearylamidase, cystinearylamidase), were detected particularly higher in LDTM8102 as compared to other strains of *Bifidobacterium*. Interestingly, EFY’s top 25 metabolites contain several types of lipid metabolites, such as esters and fatty acids, which can be produced by lipase activity during fermentation (Figure 7A). In fact, there are previous studies that have reported beneficial effects of various fatty acids and lipid metabolites on skin health. [56,57]. As mentioned above, besides the major isoflavones, various kinds of phenolics, lipids, and others in EFY may have contributed, in part, to the effect of EFY. Therefore, the different profiles of metabolites produced by different microbial enzymes may have resulted in the differences in reducing sUV-induced MMP-1 production.

A wide range of phenolic compounds, such as isoflavones and procyanidins in Yak-Kong, are hydrophobic and reported to have beneficial effects on skin wrinkles [16,50]. In this study, we used 70% ethanol extracts of unfermented and fermented Yak-Kong for experiments. We compared the efficacy of MMP-1 reduction among the extracts using hot water (75 °C) and 5%, 10%, 30%, 50%, and 70% ethanol in order to confirm the optimal conditions for extracting fermented Yak-Kong. As expected, the ethanol extracts of fermented Yak-Kong more effectively inhibited MMP-1 protein secretion than hot water extracts, and the best results were obtained from the 70% ethanol extract of fermented Yak-Kong (data not shown). In this respect, other extraction methods with higher efficiency for hydrophobic compounds, such as subcritical extraction and supercritical extraction, may be useful for developing anti-photaging functional materials from fermented Yak-Kong.

The epidermis is the primary physical barrier of skin tissue to external stresses, such as sUV irradiation and pathogen infections [58,59]. Because skin keratinocytes are mainly located in the epidermis and they constitute the majority of epidermal cells, the MMP-1 secreted from epithelial keratinocytes compromises the barrier function of the epidermis resulting in sUV-induced wrinkle formation [60,61]. The HaCaT cell line, which is derived from epithelial keratinocytes from an adult human skin, is a spontaneously immortalized skin keratinocyte cell line and it contains a p53 mutational spectrum that is consistent with distinctive DNA mutations produced by sUV irradiation [62,63]. This p53 mutation causes immortalization to render primary cells more efficiently attached. Additionally, since the HaCaT cells display patterns of growth, differentiation, and response to external stresses, such as UV exposure similarly to normal keratinocytes, the HaCaT cell line is a useful alternative to primary normal keratinocytes [64]. It has been frequently used as an in vitro model to examine the molecular mechanisms and efficacy of bioactive compounds in photoaging [25,26,65,66]. Recently, an artificial 3D skin culture model comprising multiple layers of epidermis and dermis has been used as a replacement for in vivo animal models to examine photoaging. The 3D skin model mimics the structural and biochemical components of human skin, providing an extracellular matrix network, such as collagen for extracellular macromolecules and cells from the epidermis and dermis [67]. 

## 5. Conclusions

Here, we report, for the first time, that EFY has anti-photoaging effects in both human epidermal keratinocytes (HaCaT cells) and in the 3D artificial skin model. Our observations suggest that EFY is a promising potential anti-photoaging ingredient for food and cosmetics that may help prevent sUV-induced formation of skin wrinkles. EFY can attract attention as a representative traditional functional material for foods and cosmetics because Yak-Kong is one of the traditional soybeans in Korea. Nevertheless, further clinical trials are needed in order to confirm the efficacy of EFY in preventing photoaging before widespread use of EFY in food and cosmetics.

## Data Availability

Data is contained within the article.
